# Multiple non-redundant functions underlie the essentiality of lipopolysaccharide in Gram-negative bacteria

**DOI:** 10.64898/2026.10.21.760538

**Published:** 2026-10-21

**Authors:** Ryan Adam Valdez, Nina Zheng, Sarah D. Beagle, Jiawei Sun, Kerwyn Casey Huang, Petra Anne Levin

## Abstract

Lipopolysaccharide (LPS) is well known for its immunostimulatory activity and its contribution to the permeability barrier of the Gram-negative outer membrane. Yet LPS is essential for growth in nearly all Gram-negative bacteria, even under laboratory conditions where its barrier function should be less critical. LPS contributes to envelope mechanics, membrane biogenesis, and cell division, but whether any of these functions accounts for its essentiality remains unclear. Here, we systematically tested these possibilities by depleting LPS in *Escherichia coli* and *Klebsiella pneumoniae* . Osmoprotection largely preserved cell-envelope integrity but not viability, demonstrating that the mechanical contribution of LPS alone cannot explain its essentiality. Genetic perturbations that increase peptidoglycan rigidity or alter phospholipid homeostasis partially improved growth, supporting independent roles for LPS in envelope mechanics and membrane biogenesis. An unbiased suppressor screen further identified activation of the MarA regulon as a potent mechanism for surviving LPS depletion. Protection required transport by the AcrAB-TolC multidrug efflux pump, revealing an unexpected connection between efflux and cell-envelope homeostasis. Thus, LPS essentiality emerges from multiple non-redundant contributions to Gram-negative cell-envelope physiology.

## Full Text Availability

The license terms selected by the author(s) for this preprint version do not permit archiving in PMC. The full text is available from the preprint server.

